# Diffusion of counterfeit drugs in developing countries and stability of galenics stored for months under different conditions of temperature and relative humidity

**DOI:** 10.3325/cmj.2012.53.173

**Published:** 2012-04

**Authors:** Francesca Baratta, Antonio Germano, Paola Brusa

**Affiliations:** 1 Department of Science and Technology of Drug, Pharmacy Faculty, University of Turin, Torino, Italy; 2 Aid Progress Pharmacist Agreement, San Gillio, Italy

## Abstract

**Aim:**

To investigate the diffusion of counterfeit medicines in developing countries and to verify the stability of galenic dosage forms to determine the stability of galenics prepared and stored in developing countries.

**Methods:**

We purchased 221 pharmaceutical samples belonging to different therapeutic classes both in authorized and illegal pharmacies and subjected them to European Pharmacopoeia, 7th ed. quality tests. An UV-visible spectrophotometric assay was used to determine the galenics stability under different conditions of temperature (T) and relative humidity (RH).

**Results:**

A substantial percentage of samples was substandard (52%) and thus had to be considered as counterfeit. Stability tests for galenics showed that the tested dosage forms were stable for 24 months under “standard” (*t* = 25 ± 2°C, RH = 50 ± 5%) conditions. Under “accelerated” (*t* = 40 ± 2°C, RH = 50 ± 5%) conditions, samples were stable for 3 months provided that they were stored in glass containers. Stability results of samples stored in “accelerated” conditions were similar to those obtained by on site in tropical countries and could so supply precious information on the expected stability of galenics in tropical countries.

**Conclusion:**

This study gives useful information about the presence of counterfeit medicinal products in the pharmacies of many developing countries. This should serve as an alarm bell and an input for the production of galenics. We recommend setting up of galenic laboratories in developing countries around the globe.

World Health Organization (WHO) defines counterfeit drugs as medicines that are deliberately and fraudulently mislabeled with respect to identity and/or source. Counterfeit medicines include products with correct ingredients or with wrong ingredients, without active ingredients, with insufficient or excessive amount of active ingredient, or with false or misleading labeling (1).

Counterfeits can be further classified in the following sub-classes (2):

• “perfect” counterfeits: these products contain the correct active ingredient and excipients in the right amount. They are manufactured in foreign countries and illicitly imported (parallel marketing), thus resulting in an economic damage.

• “imperfect” counterfeits: these products contain the right components, with an incorrect concentration and/or formulation resulting in defective quality specifications. In the vast majority of cases, they are devoid of any therapeutic efficacy.

• “apparent” counterfeits: they are similar to the original product, but contain non-active ingredients or foreign substances. Usually, apparent counterfeits are copies of medicinal products that are not the only ones resolutive for the cure of a particular illness.

• “criminal” counterfeits: they are apparently similar to the original medicinal product, but do not contain any active ingredient and can even include harmful or toxic substances. They are usually sold at high prices and for treatment of serious pathologies. Consequences for users of criminal counterfeits can be fatal.

Counterfeit medicines are widespread in all countries around the globe and represent a major public health concern, often resulting in treatment failure, serious deterioration of the state of health, or even death (3-10).

The true extent of the problem of counterfeit medicines is not really known, due to the fact that not every case is reported by competent authorities or manufacturers. The recent figures estimate that worldwide sales of counterfeit medicines reach at least US $3.5 billion per year (4). The phenomenon affects all pharmaceuticals, ranging from drugs for life-threatening diseases (eg, diabetes) to common-use analgesics or life-style products, such as drugs for erectile dysfunction.

The problem of counterfeits is even more serious in developing countries, where custom procedures are less stringent, authorities’ controls are less effective, and the use of ineffective drugs may result in a substantial loss of public confidence in the health care system (3). The principal target of counterfeiters are life-saving drugs. This increases the risk of resulting health damage, and sometimes gives rise to events of catastrophic proportions, like in Niger in 1995 (when about 60 000 people were injected with a counterfeit meningitis vaccine) or in Haiti in 1996 (when a diethylene glycol contamination of a pediatric syrup killed more than 80 children) (11,12).

In all developing countries, principal targets are anti-retroviral drugs, antimalarics, and antibiotics, sometimes in staggering proportions: for instance, an international study published in 2004 showed that more than 53% of artesunate tablets sold in southeast Asia did not contain any active ingredient at all, with imaginable consequences on the fight against malaria in those countries (11,13).

According to the current European Legislation, the pharmacist is the only professional figure lawfully entitled to prepare medicinal products (except, of course, pharmaceutical industries). As a matter of fact, the article 3 of the European Directive 2001/83/EC states that medicinal products prepared in a pharmacy in accordance with a medical prescription (magistral formula) or in accordance with the prescriptions of the National Pharmacopoeia (official formula) are exempt from marketing authorization procedures applied to industrial medicinal products (14-17).

Nevertheless, magistral and official formulations (commonly known as “galenics” in homage to Galen of Pergamum, who is regarded as the first pharmacist engaged in the preparation of medications) are required to be prepared, labeled, and stored using standard procedures and established methods in order to ensure the quality of the finished product, which is a mandatory prerequisite for its safety and efficacy (18-21). The quality of galenic medications does not rely only on the professional ability and scientific preparation of the pharmacist, but also on several external factors, such as storage conditions, chemical and physical nature of the active pharmaceutical ingredient (API), containers, environmental conditions, and the compatibility of API with excipients.

All these factors, considered as a whole, define the use-by date of medicinal products, which must be reported on the labeling. The current legislation has decided to define precautionary validity limits for galenics depending on the nature of their dosage form, leaving to the pharmacist the option to increase these limits based on scientific data.

We believe that galenics may considerably help in improving the health care system of developing countries essentially for two reasons: the first one is that their production is quite cheap and the operative procedures to be followed are simple; the second one, most interesting and important in our opinion, is represented by the possibility to prepare medicinal products with a dosage and pharmaceutical form that can suit the local needs. Therefore, galenics could become an answer to a need, but their stability in different environmental conditions, such as those of the tropical countries, must be demonstrated to guarantee safety and efficacy of the therapies (22,23).

In the present article, we investigated the extent of the phenomenon of pharmaceutical counterfeit in developing countries. Our aim was to investigate the quality of medicines purchased *in loco* from pharmacies or from unofficial street-pharmacists. The other aim was to evaluate the use-by date of galenic dosage forms commonly prepared in pharmacies. This aim also gave us the opportunity to determine the stability of galenics prepared and stored in developing countries, particularly those with tropical or sub-saharian environmental conditions (high temperatures and relative humidity).

## Methods

### Drugs analyzed

During our research, 221 samples were purchased and analyzed; 96% of the samples were industrial drugs and 4% were galenics; these last were included in the analysis with the aim to evaluate if the quality requirements had been respected in Brazil, where galenics are sold by some pharmacies as low cost medicines for poor people.

Drugs were purchased in the following countries: Angola, Brazil, Cameroun, Central African Republic, Chad, Congo, Ethiopia, Guinea Bissau, Guinea Conakry, India, Kenya, Madagascar, Malawi, Rwanda, and Uganda. Samples were purchased both in official pharmacies and from unauthorized street pharmacists. The tested medicinal products were chosen, in agreement with local medical doctors, between those more used for endemic diseases. The active pharmaceutical ingredients evaluated in this study were above all antibiotics and anti-inflammatories, formulated as tablets and capsules, because these are the prevalently administered pharmaceutical forms (Tables 1 and 2).

**Table 1:** Therapeutic classes of total and counterfeit samples

	No. (%) of samples	
Therapeutic classes	available for analysis	counterfeit
Antibiotics	76 (34.4)	30 (29.7)
Anti-inflammatories	44 (19.9)	22 (21.8)
Antipyretics	24 (10.9)	9 (8.9)
Antimalarics	17 (7.7)	6 (5.9)
Antimycotics	13 (5.9)	9 (8.9)
Antihypertensives	8 (3.6)	1 (1.0)
Antianemics	5 (2.3)	4 (4.0)
Spasmolytics	5 (2.3)	2 (2.0)
Diuretics	5 (2.3)	1 (1.0)
Antiacids	5 (2.3)	2 (2.0)
Bronchodilators	4 (1.8)	5 (5.0)
Others	15 (6.8)	10 (9.9)

**Table 2:** Quantity of different dosage forms of the samples

Dosage forms	Samples available for analysis
Ointments	1
Effervescent tablets	2
Creams	2
Gastro-resistant tablets	3
Oral powders	4
Vials	9
Coated tablets	21
Capsules	40
Tablets	139

For each test item, the country of origin and the declared manufacturing site were noted; samples were thus subjected to the analyses reported in the Table 3. Obviously, disintegration, friability, and hardness assays were applied only to tablets. The analyses were performed in the laboratories of the Department of Science and Technology (Faculty of Pharmacy, University of Turin, Italy). Each assay was carried out using instruments and methods in compliance with the relative monograph of the Official Pharmacopoeia of the European Union (Ph. Eur. 7 ed.) (17).

**Table 3:** Assays from European Pharmacopoeia for quality and assurance control

Analysis	Method of reference	Acceptance criteria
General aspect	Visual	Posological unit integrity
Uniformity of content	Assay 2.9.6	Each individual content is between 85% and 115% of the average content (10 dosage units)
Uniformity of mass	Assay 2.9.5	Not more than two of the individual masses deviate from the average mass by more than ±10% and none deviates by more than ±20% (20 dosage units)
Disintegration	Assay 2.9.1	At the end of the specified time all of the dosage units must have disintegrated completely
Friability	Assay 2.9.7	Maximum loss of mass not more than 1%
Hardness	Assay 2.9.8	Hardness between 4 and 10 N

Standard reagents (Carlo Erba, Rodano, Italy) and labware were used. To determine the uniformity of content, a UV-visible (UV-VIS) spectroscopic method was used to quantify the active pharmaceutical ingredient in each dosage form (using a DU 730 spectrophotometer, Beckman, Milan, Italy). For each active pharmaceutical ingredient contained in the dosage forms, a suitable solvent was chosen for UV-VIS analysis. The wavelength of maximum absorbance (λ_max_), when not available in literature (24) was determined by analyzing the UV spectrum between 200 and 600 nm.

Since all these assays were mandatory to ensure the quality of a dosage form, it was sufficient to obtain an out-of-specification (OOS) value in one of these to classify it as a counterfeit medicine. Since the quali-quantitative content of excipients was unknown in most cases, it was not possible to prepare a proper blank solution for test items; anyhow, since the comparison was made within the same dosage forms, the interferences due to excipients were minimized using solvents in which they, usually lipophilic, are almost insoluble.

### Analytical method validation

Several publications, also regarding counterfeits, propose high performance liquid chromatography (HPLC) as a suitable method for medicinal product analysis (24-30). Taking into account that Pharmacopoeia (17) does not prescribe a specific analytical method for performing the content uniformity assay (simply stating that a “suitable analytical method” should be applied), we decided to use an UV-VIS spectrophotometric method. This choice was also determined by an opportunity to apply this technique in African countries, where the instruments necessary for HPLC are too expensive. In any case, in order to evaluate the equivalence between the two methods, every sample containing amoxicillin, ibuprofen, and paracetamol was analyzed using both HPLC and UV-VIS methods and the results were compared.

### Stability study

Eight galenics were formulated in seven different dosages at the laboratory of *A.P.P.A.^®^* Project, the main project of *Aid Progress Pharmacist Agreement*, a no-profit organization proposing to assist the realization of galenic laboratories in developing countries around the globe.

The *A.P.P.A.^®^* Project is usually performed in different phases, detailed on the Web site *www.progettoappa.it*. A preliminary need of the Project is to determine (through on-site sampling) the average quality of available medicines in the area, to ascertain the percentage of substandard or counterfeit drugs, and to set up a priority list with the most problematic realities on the top. Therefore, a preliminary stability study on the most common and used pharmaceutical forms was performed. When the galenic laboratory is set up, the galenics produced in the laboratory are sent to *A.P.P.A.^®^* staff in Turin for a quality check to ensure that a sufficient quality level is continuously maintained. The quali-quantitative composition of the analyzed galenics was reported in Table 4 (18,20,21). For each galenic dosage form, the complete dosage form and its API was tested.

**Table 4:** Quali-quantitative composition of tested galenics prepared in *A.P.P.A.®* laboratories

Active pharmaceutical ingredient	Dosage form	Quali-quantitative composition
**Paroxetine**	**Capsules**	**Paroxetine 20 mg** **Starch 380 mg**
**Amoxicillin**	Tablets	Amoxicillin 500 mg 250 mg excipients (see below) Mix:
		Polyethylene glycol 4000 5%
		Magnesium stearate 4.5%
		Talcum powder 4.5%
		Corn starch 32%
		Microcristalline cellulose 54%
**Hydrocortisone acetate**	Cream	Hydrocortisone acetate 0.5% in base cream
**Ketoprofen**	Gel	Ketoprofen 5% in base gel
**Dextromethorphan hydrobromide**	Oral drops	Dextromethorphan 1.5% in poliolic vehicle
**Nifedipine**	Capsules	Nifedipine 20 mg Starch 430 mg
**Fluoxetine**	Capsules	Fluoxetina 20 mg Starch 430 mg

Two different batches of samples were prepared: the first one was prepared 18 months prior to the beginning of the study and stored at “standard” conditions (as hereinafter defined). A second batch of samples was prepared at the beginning of the stability study and used as a standard to check the stability of the first batch after 18 months’ storage.

All samples of dosage forms (in a sufficient amount to allow sampling through the whole study) were stored in calibrated thermostats. Samples of the relative powdered APIs were stored at the same conditions and analyzed in parallel with finished dosage forms. Three different conditions of temperature (T) and relative humidity (RH), ie, “standard,” “accelerated,” and “stress” conditions, were applied to all samples (31,32). The “standard” conditions (*t* = 25 ± 2°C, RH = 50 ± 5%) were applied to define the stability of samples under pharmacy storage conditions accepted by the European Pharmacopoeia.

“Accelerated” (*t* = 40 ± 2°C; RH = 50 ± 5%) and “stress” (*t* = 40 ± 2°C; RH = 80 ± 5%) conditions were investigated with a double purpose: to support real-time stability data with the results coming from accelerated storage conditions and to obtain information on the expected stability of drugs in tropical climates. For samples stored in “accelerated” and “stress” conditions, both polypropylene (PP) and glass containers were used, while samples under “standard” conditions were stored only in PP containers.

Chemical stability both of dosage forms and APIs was evaluated by a quantitative assay at different time points during the overall 6-month study period. For “standard” conditions, two sampling time points were foreseen, T0 and T6. For “accelerated” and “stress” conditions, further sampling time points were foreseen on a monthly basis. Both for APIs and dosage forms, 10 samples were analyzed at each time point and the average API concentration was calculated as the arithmetic mean of all values.

We took into account “stress” and “accelerated” conditions to gather information on the prospective stability of galenics in extreme environmental conditions (eg, in tropical countries). Eventually, we compared the stability data of galenics stored in PP and glass containers. As foreseen by the pharmacopoeia, samples complied with specifications if the API content was within ±10% of the expected value.

### Analytical method

The UV-VIS spectrophotometric assay used to determine the samples stability was the same as described before (17,24,33). In addition, a calibration curve was prepared at λ_max_ for each API and the concentration in each sample at different time points was calculated using Lambert-Beer’s law (A = ϵbc).

For the analysis of APIs contained in the dosage forms, a second calibration curve was obtained by using standard solutions prepared mixing API and excipients in the same ratio used for the dosage forms. This was done in order to avoid underestimating the samples concentration due to an incomplete API extraction from the excipients matrix.

Blank solutions were prepared by dissolving appropriate amounts of excipients contained in the pharmaceutical forms in suitable solvents. Analytical samples were extracted from dosage forms using an appropriate amount of solvent and favoring the dissolution with a vortex. After centrifugation for 5 minutes at 4000 rpm, supernatants were properly diluted using the same solvent for spectrophotometric analysis. Isolated APIs were dissolved in solvent at an appropriate concentration and analyzed directly.

## Results

### Counterfeit drugs

**Figure 1:**
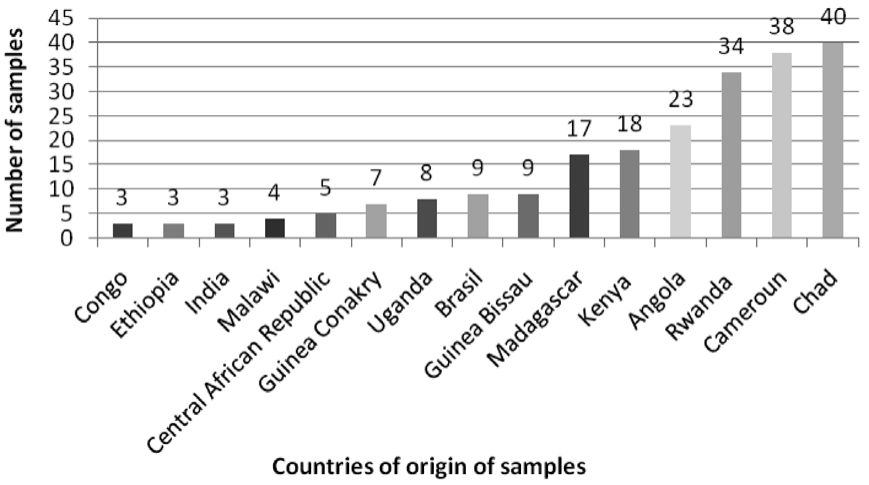
Country of origin of the analyzed drug samples.

**Figure 2:**
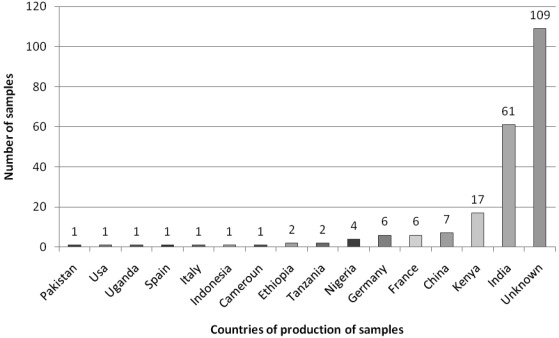
Country of production of the analyzed drug samples.

Central African Republic was excluded from the final evaluation due to an insufficient number of samples. Thus, the presented results correspond to 196 samples instead of 221 (Figure 1). The most represented manufacturing country was India (31%), but it is noteworthy that in 56% of the cases it was not possible to determine the manufacturing country (Figure 2).

**Figure 3:**
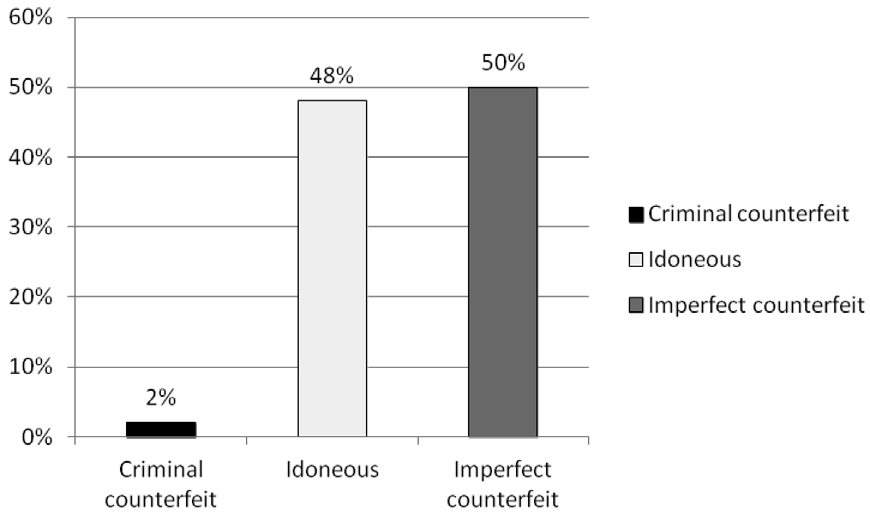
Share of counterfeit drugs in analyzed drug samples. Black bars – criminal counterfeit; white bars – idoneous; gray bars – imperfect counterfeit.

**Figure 4:**
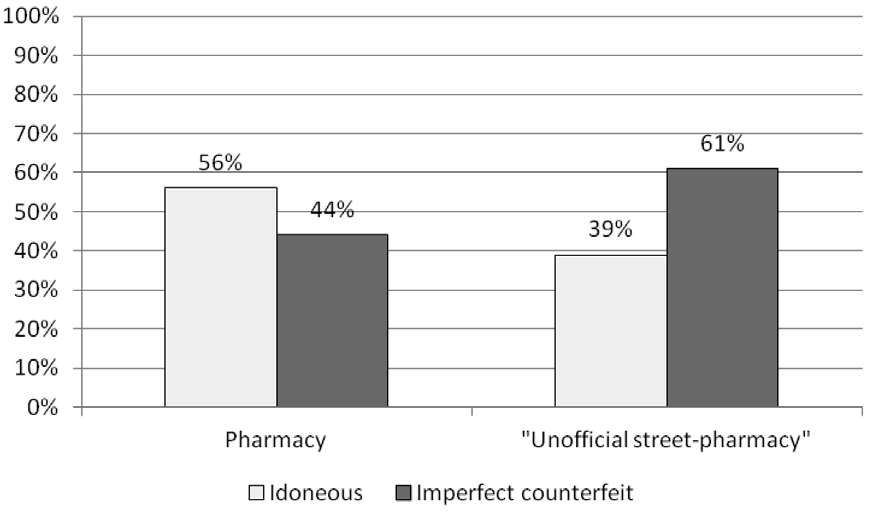
Share of counterfeit drugs sold in pharmacies vs “unofficial street-pharmacies.” White bars – idoneous; gray bars – imperfect counterfeit.

The overall percentages of samples satisfying Pharmacopoeia assays were the following: general aspect 96%, uniformity of mass 95%, uniformity of content 75%, disintegration 90%, hardness 74%, and friability 85%. Based on these results, it was possible to determine that 50% of test items were substandard drugs and even 2% were counterfeits without the presence of the declared API, ie, criminal counterfeits (Figure 3). Our results also showed that Indian drugs were often substandard: 30 out of 61 Indian samples (ie, 41.7%) showed OOS values. Only for 109 samples out of 221, it was possible to determine whether they were purchased in pharmacies (83%) or from unofficial street-pharmacists (17%). The percentage of counterfeit products was greater in the case of street-pharmacists (Figure 4).

**Figure 5:**
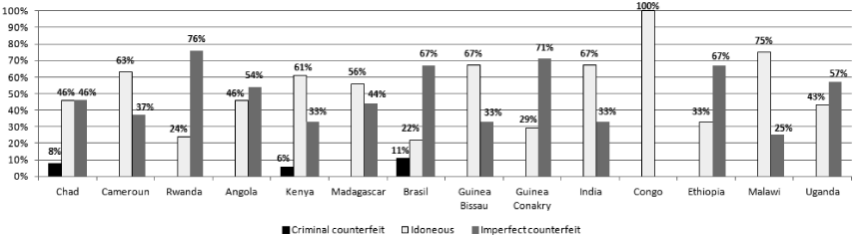
Presence of counterfeit drugs by country. Black bars – criminal counterfeit; white bars – idoneous; gray bars – imperfect counterfeit.

The overall distribution of counterfeits and substandard medicines in different countries showed that the countries mostly affected by counterfeiting were Rwanda and Guinea Conakry, where more than 70% of the analyzed medicinal products were not suitable (Figure 5). Criminal counterfeited medicines were found in Chad, Kenya, and Brazil. The results for the samples analyzed using both HPLC and UV-VIS spectrophotometry were uniform and comparable (data not shown).

### Active pharmaceutical ingredient

**Figure 6:**
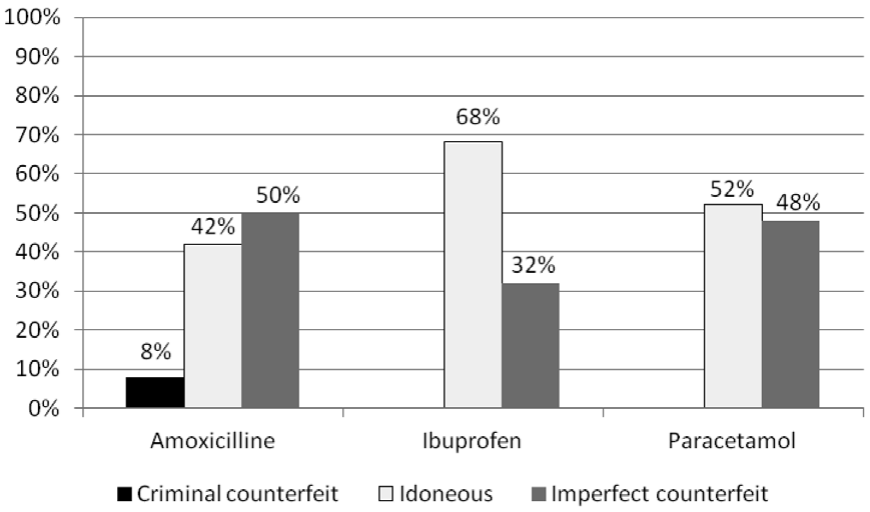
Share of counterfeit drugs in selected active pharmaceutical ingredients. Black bars – criminal counterfeit; white bars – idoneous; gray bars – imperfect counterfeit.

We stratified the results to evaluate the percentage of counterfeit drugs concerning the three most represented active pharmaceutical ingredients in our samples, ie, amoxicillin, ibuprofen, and paracetamol (Figure 6).

Amoxicillin is a semi-synthetic β-lactamic antibiotic. It is a fundamental drug in developing countries where common infections, easily treated in developed countries, often claim many lives. Its synthesis, while being quite simple, must be carried out in a controlled environment due to the sensibility of this molecule to environmental moisture. This problem is even more challenging in developing countries, where tropical climate and non-compliance with good manufacturing practices can dramatically hamper the quality of this antibiotic, during its production or during distribution and storage. We acquired 24 samples of amoxicillin (both in capsules and in tablets), mainly from Cameroun (25%) and Chad (25%). As a matter of fact, uniformity of content assay was not satisfactory in 46% of cases, with 2 samples not containing any active ingredient at all. Moreover, other 2 samples had already been broken in the blister before we even opened it and those purchased in Angola contemporaneously showed OOS results in three assays: uniformity of content, friability, and hardness.

More comforting results were obtained with uniformity of mass and disintegration assays, which were satisfied by 100% of the samples. Globally, 42% of samples were within regulatory specifications.

Ibuprofen is one of the most important non-steroidal anti-inflammatory drugs, widely used to treat inflammatory states. Nineteen ibuprofen-based drugs were analyzed during our study (tablets and capsules), mainly from Cameroun (37%) and Chad (16%). Uniformity of mass test showed that all samples satisfied the test; uniformity of content gave also good results (95% of samples within specifications). Disintegration and friability gave worse results (respectively, 11% and 16% of OOS samples). Only one sample showed substandard results on hardness assay. Sixty eight percent of samples were idoneous

Paracetamol is one of the most common-use drugs, used as an analgesic and antipyretic. Twenty-one samples of paracetamol-based medicines (tablets) were purchased, mainly in Cameroun (29%) and Chad (19%). Uniformity of content showed that 81% of samples were within specifications; friability test showed that 52% of the samples complied with European Pharmacopoeia, 7 ed. requirements, and 19% of samples did not pass the hardness assay. Fifty-two percent of samples were idoneous.

### Stability study

In the laboratory of *A.P.P.A.^®^* Project, the samples of different dosage forms were prepared as galenics with the aim to analyze their quality in different experimental conditions that simulated tropical countries weather. Seven different galenic dosage samples and their APIs were stored for 6 months in standard conditions in PP containers. Results obtained after 6 months storage showed that all galenic dosage forms and the respective APIs could be considered stable at these conditions (active substance assay within ±10% of nominal value) (Table 5). Comparison of the results obtained from the analysis of the two batches of samples (prepared respectively 18 months before the study and at the beginning of the study) 6 months later demonstrated that even samples prepared 24 months before did not present any degradation or alteration. Analysis in different environmental conditions (“accelerated” and “stress”) were in the same way performed both on samples prepared at the beginning and 18 months before the start of the study, and the results were comparable regardless of the storage time.

**Table 5:** Stability of samples stored under “standard” conditions for 6 months*

	Absorbance variation (%)	
	active pharmaceutical ingredient	galenic
Paroxetine	-1	-5
Amoxicillin	-0.3	-6
Hydrocortisone acetate	-0.6	+4
Ketoprofen	-1	-3
Dextromethorphan hydrobromide	-4	-8
Nifedipine	-0.3	-0,4
Fluoxetine	+0.1	-3

*Results are reported as percentual variation of absorbance values (arithmetic mean of 10 analyses) between sample concentration and standard.

Under “accelerated” conditions, samples were stored for 6 months at *t* = 40 ± 2°C, RH = 50 ± 5% in PP containers. Sampling and analysis were performed on a monthly basis. Due to the fact that the results after 3 months showed marked degradation both of API and galenic dosage forms, the study was interrupted at this time point (Table 6). A similar degradation pattern emerged under “stress” conditions (*t* = 40±2°C; RH = 80±5%; data not shown).

**Table 6:** Stability of samples stored under “accelerated” conditions*

	Absorbance variation (%) for		
	T1 (30 d)	T2 (60 d)	T3 (90 d)
**Active pharmaceutical ingredient:**			
paroxetine	+1	-4	+8
amoxicillin	-2	-11*	+17*
hydrocortisone acetate	-11*	-2	-37*
ketoprofen	-13*	+1	+22*
dextromethorphan hydrobromide	+11*	+23*	+25*
nifedipine	-7	-31*	+10
fluoxetine	-2	-57*	-53*
Galenics:			
paroxetine	-25*	-28*	-48*
amoxicillin	-3	-11*	-28*
hydrocortisone acetate	-8	+16*	+26*
ketoprofen	-13*	+2	+39*
dextromethorphan hydrobromide	-26*	+21*	+22*
nifedipine	-58*	-62*	-65*
fluoxetine	+22*	-56*	-56*

*Results are reported as percentual variation of absorbance values (arithmetic mean of 10 analyses) between sample concentration and standard. Out-of-specification values are identified with an asterisk.

Interestingly enough, in most cases the percentual variation of absorbance between samples and standards was found to be positive, pointing out the absence of API degradation.

Different causes were hypothesized to explain this peculiarity: for instance, the high temperatures might have damaged the PP containers, allowing moisture to degrade the preparation and to alter the UV-VIS analysis. Significantly, the absorbance increase was especially considerable at absorption wavelengths of water.

Alternatively, PP containers might have released leachable substances which might have interfered with the analysis. This last hypothesis was supported by the fact that PP containers stored under “accelerated” conditions showed color change after 40 days.

To confirm these hypotheses, a new subset of UV-VIS analyses was designed as follows:

• water samples were stored for 3 months in PP or glass containers under “accelerated” conditions;

• API and galenic dosage forms were stored for 3 months in glass containers under “accelerated” or “stress” conditions. Only 4 of 7 APIs and their dosage forms were evaluated (paroxetine, hydrocortisone acetate, ketoprofen, and nifedipine).

The UV-VIS analysis of water samples stored in PP containers under “accelerated” conditions resulted in the identification of peaks not present in water samples stored in glass containers under the same conditions. The increase in the intensity of these peaks (possibly due to the contamination of extractable and leachable substances from the PP container) was time-dependent and their absorption wavelengths corresponded to the peaks revealed in the stability study. These results suggest that in a hot and moist environment (such as in tropical countries), standard PP containers are not a suitable choice to store medicinal products and that glass containers are to be preferred.

Results obtained under “accelerated” and “stress” conditions on galenic samples stored in glass containers for 3 months showed that all samples stored under “accelerated” conditions complied with the acceptability criteria after 90 days (APIs and dosage forms), while “stress” conditions resulted in substantial degradation both of APIs and dosage forms after 90 days (Table 7). Remarkably, the spectrum results were more altered for the dosage forms than for the isolated APIs.

**Table 7:** Stability results under “accelerated” conditions (AC) and “stress” conditions (SC) for samples stored in glass containers*

	Absorbance variation (%)							
	paroxetine		hydrocortisone acetate		ketoprofen		nifedipine	
	AC	SC	AC	SC	AC	SC	AC	SC
**Active pharmaceutical ingredient:**								
**T1 (30 d)**	+3	+5	+0,5	+1	-1	+14*	+1	-42*
**T2 (60 d)**	+3	+17*	+0,7	+13*	-1,5	+23*	+0,6	-47*
**T3 (90 d)**	+2.5	+24*	+0.7	+19*	+1	+32*	+1.5	-52*
**Galenics:**								
**T1 (30 d)**	-1	+6	+0,2	+7	-0,3	+34*	-3	-64*
**T2 (60 d)**	-4	+33*	+0,6	+18*	-0,6	+91*	-2	-72*
**T3 (90 d)**	-4.5	+42*	+0.8	+59*	-0.9	+99*	-2.5	-83*

*Results are reported as percentual variation of absorbance values (arithmetic mean of 10 analyses) between sample concentration and standard. Out-of-specification values are identified with an asterisk.

## Discussion

Counterfeit medicines are one of the most problematic issues in developing countries, where absence of controls and an adequate pharmacovigilance system causes difficulties in revealing and monitoring the phenomenon and its effects among the population. Our results were in accordance with international data (9,34,35); 2% of tested samples could be defined as criminal counterfeits and about 50% as imperfect counterfeits, meaning that these drugs could not serve their purpose as intended. We used UV-VIS spectroscopy to quantify the active pharmaceutical ingredient in each dosage form and to carry out the uniformity of content assay. UV-VIS spectroscopy was found to be a suitable method to comply with European Pharmacopoeia, 7 ed. provisions; moreover, it can represent the ideal analytical method to be used routinely in developing countries, due to its reduced costs and simplicity. It is important to note that European Pharmacopoeia, 7 ed. does not define a univocal method to be used for content uniformity assay, but refers only to a “suitable” method. Cross-check analysis using a HPLC method demonstrated the consistency of UV-VIS results.

The main target of counterfeiters are highly-priced, life-saving drugs and this trend is likely to be maintained (3-6,35).

A substandard antibiotic can be even more dangerous than other drugs because it can promote the onset of bacterial resistances. From the collected data, it is evident that (even taking into account degradation caused by environmental conditions) amoxicillin is widely subject to counterfeit; a possible explanation for this is the high price of this active ingredient (averagely €120/kg), which can make it a desirable target for counterfeiters. The minor presence of counterfeit ibuprofen (averagely €30/kg) and paracetamol (averagely €26/kg) if compared to amoxicillin (averagely €120/kg) can be explained by their lower prices.

The main limitation of our study is the small number of samples; nevertheless, since our overall findings are in good agreement with more vast international studies, we deem that useful considerations may arise also from small-sized surveys such as ours.

In accordance with WHO assessments, India was the first exporting country among those examined in the present study, with 61 samples (about 50% of which were counterfeits). This prominent role of India in the production of counterfeits could be a result of several different causes, among which is the permissive legislation and inefficient judiciary system, absence of qualified supervising staff, and widespread corruption (3).

In the EU, Good Manufacturing Practices are enforced by European Medicinal Agency (EMA), while for Asiatic manufacturers adherence to Good Manufacturing Practices requirements is more inconsistent, due to the fact that competent authorities audits are often insufficient (36-38).

The second aim of the present study was the stability study of common galenic medications and the assessment of their use-by date under different environmental conditions..This study showed that all the tested dosage forms and active substances were stable for 24 months under “standard” conditions (active substance assay within ±10% of nominal value) in PP containers. Application of “accelerated” and “stress” conditions caused a significant degradation if dosage forms were stored in PP containers, but results were within specifications after 3 months if samples were stored in glass containers in “accelerated” conditions. Since it is common practice to consider that 1 month storage under “accelerated” conditions equals to 4 months storage at room temperature, from the results at 3 months a stability at ambient conditions of at least 12 months can be extrapolated. In our opinion, this could represent a good goal because the expiration date usually proposed for galenics in the absence of experimental data are very short.

The investigation proved that moisture is the principal cause of chemical degradation of these APIs and that, as a consequence, any step taken to reduce the contact between environmental moisture and the galenics is likely to result in a substantial increase in their stability. Eventually, stability results on APIs suggest that the excipient is usually the most sensible component of galenic preparations, in a degree proportional to its hydrophilic characteristics. This hypothesis is supported by the poor stability of ketoprofen gel, for which degradation rate was equal to 91% after only two months, if compared to the greater stability of hydrocortisone cream (degradation rate 18% after two months). The above considerations are suggestive of a role of hydrophilicity of excipients in determining the susceptibility to moisture-induced degradation.

Based on these stability data, a minimum use-by date equal to 24 months under “standard” storage conditions (*t* = 25 ± 2°C, RH = 50 ± 5%) can be proposed for the dosage forms investigated during the study. “Accelerated” results proved that this minimum period can be safely increased according to current European Medicinal Agency guidelines. These guidelines give very precise information about medicine’s expiration date, which depends on environmental conditions (T and RH) applied during the stability tests and on their duration (31,32).

Further studies should be designed to define the limit shelf-life for these dosage forms, but based on our results it is possible to hypothesize that current limits set out by Italian Pharmacopoeia (maximum 6 months) (22,23) are rightly precautionary but excessively restrictive (at least limited to the preparations investigated in the present study).

Also in the study of galenics, we demonstrated that UV-VIS spectroscopy results were comparable and uniform to those obtained by HPLC analysis. We deliberately chose to use an easy, affordable analytical method such as UV-VIS spectroscopy in order to mime the work conditions of chemist laboratories and to supply pharmacists with useful directions to check the stability of their own galenics.

Our results allow us to prolong the shelf-life of galenics if the procedures used to prepare and store the different dosage forms follow general principles. It is important to note that in developing tropical countries PP containers are not suitable both for home and pharmacy storage if the space is not air-conditioned; glass is to be preferred in hot and moist climates due to the fact that it is able to guarantee a longer stability to dosage forms contained.

Irrespective of what type of test is applied (test in “standard” or “accelerated” conditions), based on obtained data and provided that galenics are prepared using written standard procedures and starting materials of sufficient quality, it is reasonable to increase the use-by date of dosage forms compared to that suggested by Pharmacopoeia. This represents an important step for the organization of the galenic laboratory. It would be easier to plan preparation activity satisfying both the medicine request of the hospital and the operative time of the technicians engaged.

In conclusion, the achieved results highlighted the importance of our Project to help the production of galenics complying with quality requirements, especially in those countries where people are most vulnerable to counterfeit drugs, which threaten their health and their faith in modern medicine.
